# Notes and News

**Published:** 1901-02-23

**Authors:** 


### NOTES AND NEWS.

### HOSPITAL MEETINGS.

West End Hospital, Welbeck Street.—Annual general meeting, Friday, February 22nd, 4 p.m.

Great Northern Central Hospital [illegible] meeting, Friday, February 22nd, 5 [illegible]

Queen Charlotte's [illegible]—Annual general meeting, Monday February 25th, 3 p.m.

Tottenham Hospital.—Annual general meeting, Tuesday, February 26th, 5.15 p.m.

East London Hospital for Children.—Annual general meeting, Wednesday, February 27th, 2.30 p.m.

Royal Hospital for Children and Women.—Annual general meeting, Wednesday, February 27th, at the Mansion House, 3 p.m.

Cancer Hospital.—Annual general meeting, Wednesday, February 27th, 4 p.m.

Seamen's Hospital.—Annual general meeting, Wednesday, February 27th, 3 p.m., at the Institute of Chartered Accountants, Moorgate Place, E.C.

Middlesex Hospital.—Quarterly Court, Thursday, February 28th, 12 noon.

King's College Hospital.—Annual general meeting, Thursday, February 28th, 4 p.m.

National Orthopædic Hospital.—Annual general meeting, Thursday, February 28th, 4 p.m.

---

By the will of the late Mr. J. W. O. Howes the Cheltenham General Hospital will receive upwards of £11,000.

The dinner of the Royal Medical Benevolent College has been postponed to July 9th, when Lord Rosebery will preside.

Dr. J. Jameson has left in his will £1,000 to the Hull Infirmary, the same sum to the Lloyd Cottage Hospital, Bridlington, also £500 to the Hull and Sculcoates Dispensary.

The Earl of Dysart has promised £500 towards new buildings at the Liverpool Hahnemann Hospital, provided others will give the remaining £2,500 necessary. He also promises £1,000 towards removing the debt of £6,000 if the remaining £5,000 is forthcoming by August, 1901.

A decision of the United States Commissioner on immigration which has been recently given is of a novel and drastic character. He has decided that tuberculosis is a disease which may subject the patient to quarantine. If this restriction were enforced upon those who visited our colonies or dependencies for purposes of health, much consternation would be created, and yet it is in respect to new countries that stringent regulations would be most valuable.

A report on yellow fever has been received from the late Dr. Myers and Dr. Murham by the Liverpool School of Tropical Diseases.

---

Professor Dussaud, of the Paris Psychological Institute, has been occupied for some time in perfecting apparatus invented by him whereby the blind are able to receive impressions of movements, such as are represented by the cinematograph. Thus the idea of the movements of waves, currents of wind, &c., become realisable to the blind.

---

The news coming from the Cape as to the presence of bubonic plague varies. The danger from the contagion from the rats is contradicted, as those that have been found dead are stated not to have died of the disease. The dock labourers who fled from their work in panic are gradually coming back. In Bombay the number of cases is still very large.

---

On the arrival of the *Egypt* at Plymouth the Port Medical Officer examined the five hundred passengers on board, a death from bubonic plague having occurred when the ship was at Bombay. The examination proving satisfactory, the *Egypt* proceeded to London. The dangers to Plymouth are minimised by the fact that the vessels arriving from the East do not come alongside of the pier, and thus the introduction of [illegible] rats is prevented. Further, doubtful cases are taken to vessels adjacent and are not landed.

---

Resource in methods of advertisement appear to be inexhaustible. The newspapers recording the ailment under which a public person is suffering contains a whole field of suggestion to the agile advertiser. Mr. Jones suffering from an attack of gout wonders how it is he so opportunely receives a circular offering him an infallible cure for his malady; whilst Mrs. Jones, whose portrait is shown in an illustrated paper, has a sheaf of useful hints showered upon her as to replenishing her scanty locks by the innumerable devices of the obliging hairdresser, both by means of additional locks or some marvellous hair restorer. The vendors of quack remedies know their public, and it would surprise the public were a list of the honoured names of those who have fallen victims to these baits to be published.

---

The name of the English hospital in Paris—the Hertford Hospital—is quite familiar, but to many its origin is forgotten or unknown. It was called after the Marquis of Hertford, in whose memory it was founded by the late Sir Richard Wallace. Sir Richard placed it under the management of the British Government in 1891. It was endowed by the late Lady Wallace in accordance with her husband's wishes. Practically the Government had not control until last year, and since then the Committee of Management have been engaged in renovations and alterations in the arrangements and the entire administration. A large sum of money has been spent. The Medical Staff consists of Dr. Paul Porier, surgeon; Drs. Faure, Miller, Daniel Anderson and Leonard Robinson, physicians. Dr. Arthur Clark, late house-physician at Guy's, is house-surgeon; Dr. G. Bull is ophthalmic surgeon; Mr. Edward Neech, dentist; and Dr. W. Douglass Hogg, dispenser. There is a secretary, matron, and staff of six nurses. The number of beds is forty, divided equally between the two sexes. The hospital has just been re-opened.

